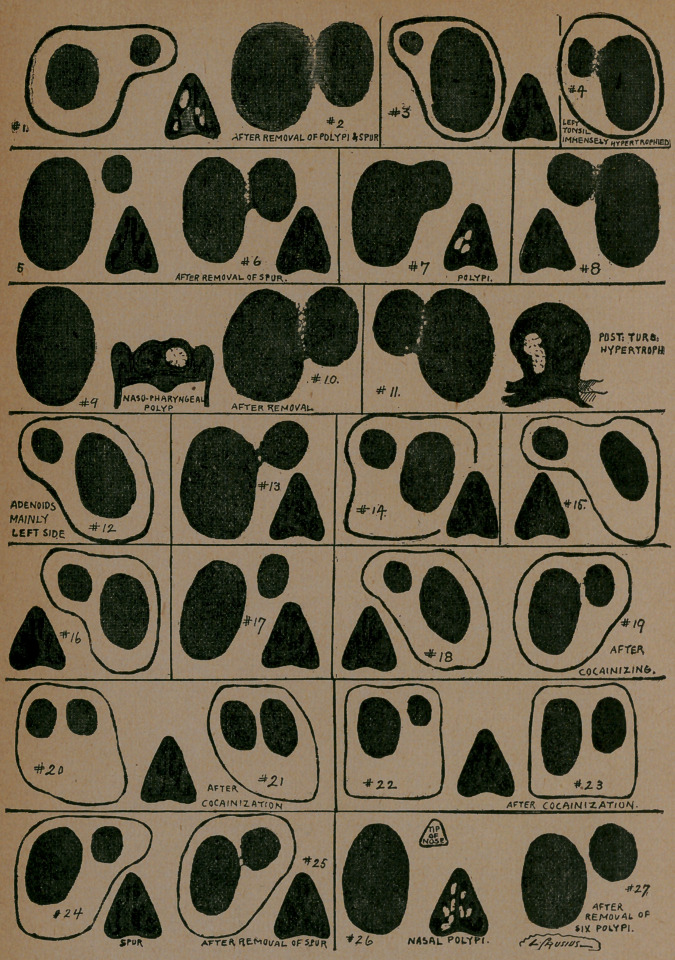# The Mirror Test for Nasal Obstruction

**Published:** 1895-01

**Authors:** Hanau W. Loeb

**Affiliations:** St. Louis, Mo.; Professor of Diseases of the Nose and Throat in the Marion-Sims College of Medicine and the Woman’s Medical College; Surgeon to the Nose and Throat Department of the Rebekah Hospital, Missouri Pacific Hospital, Woman’s Hospital, M., K. & T. Hospital, Grand Ave. Free Dispensary, Etc.; 321 North Grand Ave.


For Texas Medical Journal.

### THE MIRROR TEST FOR NASAL OBSTRUCTION.

BY HANAU W. LOEB, A. M., M. D., ST. LOUIS, MO.

Professor of Diseases of the Nose and Throat in the Marion-Sims College of Medicine and the Woman's Medical College; Surgeon to the Nose and Throat Department of the Rebekah Hospital, Missouri Pacific Hospital, Woman's Hospital, M., K. & T. Hospital, Grand Ave. Free Dispensary, Etc.

Read before the Missouri State Medical Association, 1894.

IN the *Archiv fur Laryngologie*, Vol. I, page 175, there appeared an article by Zwaardemaker, of Utrecht, which first called my attention to the possibility of determining the presence of nasal obstruction through the agency of the film of moisture deposited by the expired air upon the mirror. Since this, I have made numerous observations in the direction of making this little experiment of practical utility. Zwaardemaker has well shown the relation between the size of the film and the obstruction. If the obstruction is great, the quantity of air which is able to pass through the corresponding side of the nose will be lessened, and therefore the water in the expired air is comparatively small in amount. As the result of this, the film deposited upon the mirror will not be nearly so great as that which comes from the nostril which presents no interference with the normal supply of air expired. Where the nasal air space is abnormally large, the reverse naturally occurs, so that one can readily determine through the medium of the vapor image upon the mirror which side is obstructed and which side is free.

After making a number of observations, it is easy to determine, almost without comparison if the slightest obstruction exists. Zwaardemaker calls attention to the utility of mirrors held in a horizontal direction at the level of the chin, lower lip and upper lip. I have been unable in this way either to obtain or to verify his results to any considerable extent.

My common practice is to have a patient stand before a large mirror, with the head thrown well back, and the nose close to the mirror or touching it. He is then directed to expire rather forcibly through the nose, his mouth being closed.

If the mirror be not too warm, the breath image will be very readily observed. The film coming from the obstructed side of the nose will be small in size, and will disappear more quickly than the corresponding film from the other side of the nose. In fact, to my mind, the rapidity of the disappearance of the image is quite as certain an indication of obstruction as the size of the film itself. This however must be noted: the colder the mirror, the more permanent will be the image, though under all circumstances it will be observed that the film which results from the greater obstruction will always disappear more quickly than that which results from a more pervious nasal fossa.

This method of nasal examination appeals not only to specialists, and general practitioners, but also to the patient, for by this means he obtains a visual indication of his condition, and he becomes more willing to submit to such operative procedures as will have the tendency to equalize the nasal cavities. A physician too may note the progress of the case that he is treating by making a record of the breath images at different times in the course of treatment. Out of the many observations which I have had occasion to make I am able through the kindness of my friend Dr. Crusius, to present drawings of some of the most interesting cases. The value of this method of examination will become most apparent by closely studying the individual figures.

Figure 1 exhibits the nasal breath film resulting from the presence of polypi and a spur upon the right septum. The right side being the most obstructed, of course shows its condition by a smaller film upon that side as compared with the other. The section as represented is made as if the patient is sitting in front of the operator. The breath picture is copied directly from the mirror, hence in this film, as well as in all the remaining diagrams taken from the observation through the anterior nares the image represented will be the reverse of the diagram showing the nasal condition, so that to translate the figures

#1

#2

AFTER REMOVAL OF POLYPI & SPUR

#3

#4

LEFT TONSIL IMMENSELY HYPERTROPHIED

5

#6

AFTER REMOVAL OF SPUR.

#7

POLYPI.

#8

#9

NASO-PHARYNGEAL POLYP

#10.

AFTER REMOVAL

#11.

POST. TURB. HYPERTROPH

ADENOIDS MAINLY LEFT SIDE

#12

#13

#14

#15

#16

#17

#18

#19

AFTER COCAINIZING.

#20

#21

AFTER COCAINIZATION

#22

#23

AFTER COCAINIZATION.

#24

SPUR

#25

AFTER REMOVAL OF SPUR

TIP OF NOSE

#26

NASAL POLYPI.

#27

AFTER REMOVAL OF SIX POLYPI.

properly the right of one must be understood to represent the left of the other.

Figure 2 shows the result of the removing of the spur and the polypi. The left image is somewhat increased; very properly so, because a greater amount of obstruction was removed from the right nostril than the left.

Figure 3 exhibits the sort of a film which is quite common in cases where a deflection and spur make a decided obstruction. The small air space represented in the left nostril of the diagram makes itself manifest in the small breath film resulting from the nasal expiration through the affected side.

The only case where I was able to get any difference in the nasal breath film from the hypertrophied tonsil is exhibited in Figure 4. In this case the left tonsil was very considerably hypertrophied while the right tonsil was hardly visible.

In Figures 1, 3, and 4, the external dark line exhibits the appearance of the film as it first appears. As the film disappears it is observed to assume the shape and position indicated by the dark surface within the outer line. This is a very important point, and is almost always observed, whenever the patient expires too forcibly. Sometimes both sides appear about the same, until the film begins to disappear, when it separates and exhibits the different patency of the two sides.

Figure 5 is quite similar to Figure 3, the main difference being that the spur in this case is on the right side, instead of the left, when consequently the images are reversed.

Figure 6 shows the same case after the removal of the septar spur. The diagram of the nose shows the improvement that was effected by the operation. In Figure 7 the polypi and the deflected septum exhibit the effects of their presence in the accompanying representation of the film resulting from the application of the mirror test.

In Figure 8, the marked thickening of the septum and hypertrophy of the inferior turbinated suffices to make a decided difference between the breath image of the two sides.

Figure 9 represents a most beautiful illustration of the efficiency of this method of examination. The patient was sent to me by Dr. Barck, who found it impossible to introduce the Eustachian catheter through the right nostril. Before any further examination was made of the nose, or naso-pharynx, the patient was directed to breathe upon the mirror, the result being a decidedly large breath image upon the left side, and absolutely nothing upon the right side. The accompanying diagram shows what further examinations revealed. The polypus which completely obstructed the right posterior nares was removed by the writer's electro-cautery snare, after which the mirror test was again applied with the result as shown in Figure 10. Certainly no more practical illustration of the worth of this method could be shown.

In Figure 11, the right nostril is obstructed by considerable hypertrophies of both the middle and inferior turbinateds, and as a matter of course the corresponding side shows a greater obstruction than the other side when a comparison of the breath images is made.

Figure 12 shows one of the few cases where a difference between the two sides was shown in the condition known as adenoids of the naso-pharynx. In this case the adenoid growth descended quite low upon the left side, filling a considerable portion of the fossa of Rosenmueller and passing down well over the left turbinateds.

Figures 13, 14, 15, 16 and 17, exhibit various degrees of deflection of the septum, in two cases accompanied by ridge. They are represented here for the purpose of further illustrating the value of this means of examination.

Figure 18 is taken from a case of acute rhinitis, in which there is also a great deflection of the septum of the right side. The left side, however, was more obstructed on account of the greater amount of swelling from the acute inflammatory process. We therefore find from the mirror test, that the right side exhibits a greater degree of patency than the left side. Upon the cocainization quite the reverse is exhibited. The swelling upon the left side was of course very materially decreased. The swelling upon the right side increased somewhat between the time of the first test and the second test, and as a result the left film is very greatly enlarged while the right was somewhat reduced.

In 20 and 21 are shown the result of cocainization in a case of chronic rhinitis, in which there was considerable swelling. Cocaine had the effect of increasing the size of both films.

In Figures 22 and 23, we see under the same circumstances both films increased in size.

Figure 24 shows the expiratory effect of a decided spur upon the right side of the septum, and Figure 25 shows the decided improvement which was not noticeable to the patient himself after the removal of the spur.

Figure 26 represents the results of this examination in the patient suffering from polypi upon both sides, completely obstructing the right side of the nose, while the left side was more or less open, inasmuch as the polyp was considerably smaller and higher up. The polypi on the right side was removed in a few minutes at the first sitting by the use of the writer's electro-cautery snare, and the mirror test was applied immediately thereafter with the result as shown in Figure 27. This was quite possible, inasmuch as since the polypi was removed by means of the electro-snare, no bleeding whatever resulted.

In conclusion, I desire to commend this method of examination to the members of the Association, feeling sure that it will only be productive of much good, and will throw considerable light upon the subject, which at best does not receive proper attention among the general practitioners of to-day.

321 NORTH GRAND AVE.

## Figures and Tables

**Figure f1:**